# Effect of patient factors and procedure on proximal tibia fracture recovery - A retrospective analysis

**DOI:** 10.12669/pjms.40.10.9154

**Published:** 2024-11

**Authors:** Tashfeen Ahmad, Zehra Abdul Muhammad, Fatima Zehra

**Affiliations:** 1 Tashfeen Ahmad, FCPS, PhD. Assistant Professor, Departments of Surgery and Biological & Biomedical Sciences, The Aga Khan University, Karachi, Pakistan; 2 Zehra Abdul Muhammad, MBBS, MSc. Senior Instructor, Department of Surgery, The Aga Khan University, Karachi, Pakistan; 3 Fatima Zehra, MBBS. Jinnah Sindh Medical University, Karachi, Pakistan

**Keywords:** Proximal tibia fractures, Gender, Age groups, Open Reduction Internal Fixation, External fixator

## Abstract

**Objective:**

Tibia plateau fractures account for 1-2% of all fractures and might adversely affect the knee joint. The current research aimed to evaluate the effect of surgical procedures, age, and gender on proximal tibia fracture functional outcomes.

**Methods:**

The present data for the retrospective analysis was obtained from an observational longitudinal cohort trauma registry study, initiated at a tertiary care hospital in June 2015. A total of 51 isolated tibia plateau fracture patients were routinely treated with open reduction internal fixation or external fixator and assessed for functional outcomes till twelve months by the Rasmussen scoring scale. Patients were divided into five age groups. The functional outcome association to gender, age groups, and surgical procedures was assessed by the Fisher Exact test and logistic regression analysis.

**Results:**

The mean age of the total 51 patients was 40±11 years. Patients operated with Open Reduction and Internal Fixation (74.5%) or with an external fixator (25.5%). Road traffic accidents were the common mechanism of injury. There were non-significantly different functional outcomes observed among age groups or between surgical procedures at all follow-ups but significantly different between genders at three-month follow-ups. Males recovered better than females (p=0.02).

**Conclusion:**

Tibia Plateau fracture patients respond almost equally to both external fixation and Open Reduction Internal Fixation procedures. Age does not influence the outcome. However, males had better functional outcomes at three months post-surgery indicating that gender could affect the outcomes. Further exploration might assist in planning gender-based proximal tibia fracture treatment strategies for optimum outcomes.

## INTRODUCTION

Tibial plateau fractures account for about one percent of all fractures and the global incidence reported is 10.3 per 100,000 people yearly.^1^ The average age is 52.6 years for patients who suffer from tibial plateau fractures.^2^ High-energy trauma is the predominant cause of this fracture.^3^ tibial Plateau Fractures usually increase the risk of osteoarthritis with an incidence of 13%-83%, disability, and deleterious effects on quality of life.^4^,^5^ The incidence of tibial plateau fractures is bimodal, with males less than 50 years old enduring tibial plateau fractures secondary to high-energy trauma, whereas females above 70 years of age sustain tibial plateau fractures secondary to low-energy trauma.^2^,^6^

Schatzker and AO classification is the most used to classify Tibial Plateau Fractures.^7^,^8^ The tibial plateau fracture treatment modality is chosen based on the type of fracture, associated injuries, surgeon’s experience and choice, age, bone quality, co-morbidities, and extent of mobility.^9^,^10^ Simple tibial plateau fractures are usually managed conservatively, while complex are treated with operative procedures to achieve a stable, and well-aligned joint with better outcomes.^9^,^11^ Commonly used surgical procedures include Open Reduction and Internal Fixation (ORIF), external fixation, and closed reduction and internal fixation (CRIF). Initially, ORIF was the most acceptable treatment for tibial plateau fractures. However, this procedure carries a risk of skin necrosis and infection with large surgical incisions and soft tissue dissection.^11^-^14^ Over the years, external fixation has been preferred as the procedure causes minimal soft tissue damage, and blood loss, allows early weight bearing post-operatively, and after fracture healing, there is no risk of implant left inside.^11^-^13^

In several studies, tibial plateau fracture outcomes were assessed after ORIF and external fixators procedures either individually or compared both. Some literature shows that on performing ORIF, there was no statistically significant difference in clinical and functional outcomes among age categories. They found that the elderly patients performed almost equally well post-ORIF procedure as the younger patients and that older age is not a risk factor for poor outcomes.^15^-^19^

In a quasi-experimental study conducted by Hassan HU, the association of functional outcome to age, gender, BMI, and Diabetes Mellitus was evaluated after three months of Ilizarov external fixator procedure in treating closed proximal tibial plateau fractures. Using Jensen’s grading system, functional outcome was affected by age and gender, with young and male patients benefitting more from the procedure than other populations.^20^ A study conducted on external fixator procedure showed excellent to good functional outcomes in patients with tibial plateau fractures with no significant difference in the outcome based on age and gender, thus, concluding this procedure as a favorable treatment modality for tibial plateau fractures.^21^

A few retrospective studies that compared outcomes of patients sustaining tibial plateau fractures who were managed with either ORIF or external fixation showed no statistically significant difference in the functional and clinical outcomes for both procedures (p≥0.05) and variables like age, gender, comorbidities, and trauma energy level did not affect the outcomes. The study concluded that tibial plateau fractures have similar outcomes following both ORIF and external fixation.^22^,^23^

The current study aimed to assess the effect of age and gender variables on recovery and functional outcomes from trauma-associated tibial plateau fractures after ORIF or external fixation procedures. There are normal anatomical differences between the major joints of males and females. Gender-based differences in the normal range of motion may affect the post-fracture recovery and function but very scarce data is available on functional outcomes differences in age groups and gender after tibial plateau fracture treatment with ORIF and external fixator. The results might assist orthopaedic surgeons in planning age and gender-specific treatment approaches for improving patient quality of life, minimizing disability, and achieving an optimal functional outcome.

## METHODS

The observational longitudinal cohort orthopaedic trauma registry was initiated in June 2015. All patients were assessed for eligibility criteria.

### Inclusion & Exclusion Criteria:

All patients, regardless of age and gender, arriving with lower limb fractures due to trauma at a tertiary care hospital were included in the registry. Patients with stress or pathological fractures and treated with amputations were excluded. Informed consent was administered to eligible patients. The patients were followed up after treatment at 2-6 weeks, 3-6, and 12 months, and functional outcomes were assessed using validated fracture-specific scoring scales. Data were gathered from patient medical record files or online hospital systems.

A total of 84 patients arrived at the hospital with tibial plateau fractures of which 11 had open fractures (including one firearm injury) and 22 had two or multiple fractures. Current prospectively collected data derived from the trauma registry on adult, isolated, closed tibial plateau fracture patients who arrived between June 2015 to January 2020 and were routinely treated with the ORIF or external fixator, were analyzed retrospectively. Patients who had open fractures, or two/multiple fractures were excluded from the analysis to avoid possible influence on outcomes. As the trauma registry is an observational study, no intervention was done. The surgery was performed as per routine care at the discretion of treating surgeons and as per the patient’s need. A total of 51 patients were finally included in the analysis. At follow-ups, functional outcomes were assessed using the Rasmussen scoring scale.^24^

### Ethical Approval:

The study was approved from the Ethical Review Committee from the Aga Khan University (Reference numbers 3194-PED-ERC-14 - July 08, 2015, August 04, 2016, September 15, 2017; 2018-0525-540 - October 29, 2018; 2019-0525-4984 - September 19, 2019),

### Statistical Analysis:

Data were analyzed by SPSS version 19.0 software. For quantitative variables like age, mean±SD or median (IQR) were calculated and frequencies (percentage) were assessed for qualitative variables like functional outcome grades, gender, etc. Shapiro Wilk test of normality was applied to age. Patients were divided into age groups in years 16-25, 26-35, 36-45, 46-55, and 56 or above. The association of functional outcomes to age groups, gender, and procedures (ORIF and External fixator) was analyzed by the Fisher Exact test and logistic regression analysis. A *p*-value < 0.05 was considered statistically significant at a 95% confidence interval.

## RESULTS

Fifty-one isolated tibial plateau fracture patients who were treated either with ORIF or an External fixator were retrospectively analyzed. The mean age was 40±11 years. Descriptives of patient demographics, characteristics, age groups, and procedures performed are cited in Table-I.

**Table-I:** Demographics, patient characteristics, and treatment.

Age (Mean±SD)	40±11 y
***Age groups (years)***	***N (%)***
16-25	2 (4%)
26-35	17 (33%)
36-45	17 (33%)
46-55	12 (23.5%)
≥56	3 (6%)
***Gender***	***N (%)***
Male	45 (88%)
Female	6 (12%)
***Comorbidity***	***N (%)***
Depression	1 (2%)
Diabetes mellitus + ischemic heart disease	1 (2%)
Hepatitis B	1 (2%)
Hypertension	1 (2%)
Hypothyroidism	1 (2%)
Ischemic heart disease	1 (2%)
Ischemic heart disease + Hypertension	1 (2%)
Diabetes mellitus + Hypertension	3 (6%)
Diabetes mellitus	4 (8%)
None	37 (72%)
***Surgical procedure***	***N (%)***
Open reduction internal fixation	38 (74.5%)
External fixator	13 (25.5%)

N=Number of patients.

**Fig.1:**
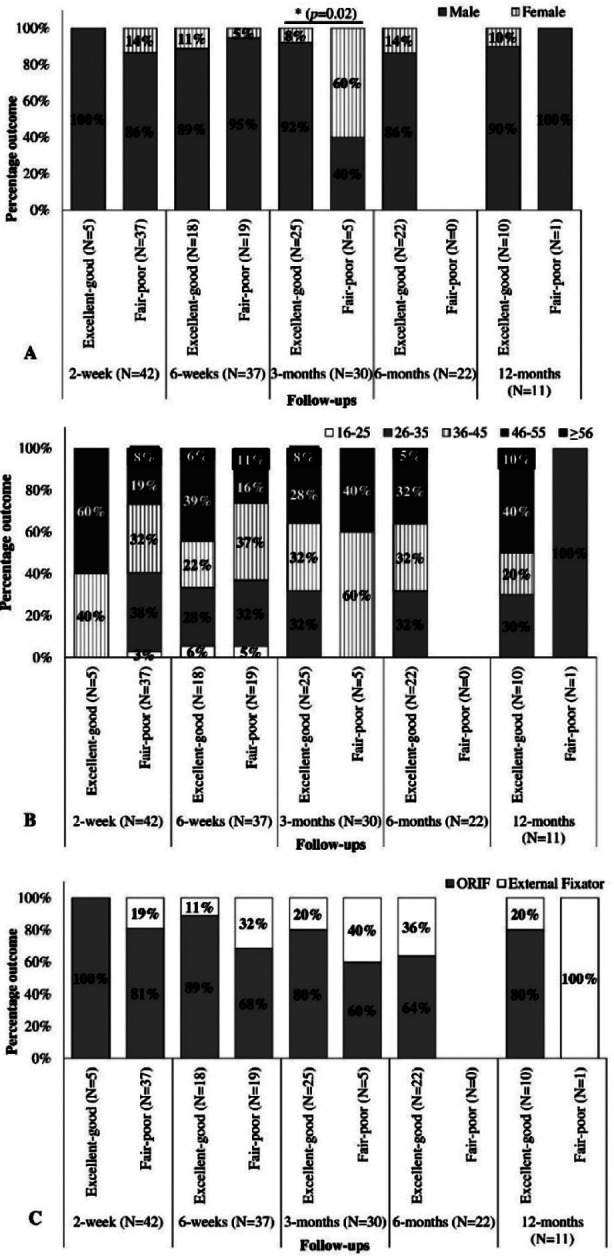
Functional outcomes at follow-ups between genders. (A), Age categories (B), and Surgical procedures (C). * = Significant difference, N=Number of patients.

On the Fisher Exact test, significant differences in functional outcomes between genders were observed at three months follow-up. Males recovered better than females at this time point (*p*=0.02, OR=0.05) (Fig.1A). A non-significant difference between functional outcomes to age groups (Fig.1B) or procedures (ORIF and External fixator) (Fig.1C) was observed at all follow-ups (*p*>0.05). The three-month outcome was reanalyzed using logistic regression analysis, showing the model was statistically significant (X^2^=6.36, *p*=0.01), explaining 32% (Nagelkerke R^2^) of the variance in procedures and correctly classifying 83% of cases. The odds of a fair-poor outcome in males were 0.058 times that in females.

On investigating the individual functional scoring scale components, it was found that the pain outcome at three months follow-up was significantly different between genders (p=0.04). Moreover, 60% of females and 40% of males lack knee extension, 60% of females and 28% of males have an incomplete range of motion while 80% of females and 44% of males were unable to walk. The stability was almost equal in both genders, (Table-II).

**Table II:** Rasmussen scoring scale subcomponent analysis for functional outcomes.

Pain	Females	males	p-value
Stabbing pain	3	3	0.0413*
No or occasional pain	2	22	

***Walking capacity***	***Females***	***males***	

Walking indoors only or in a wheelchair	4	11	0.32
outdoor short walk to normal walking	1	14	

***Extension***	***Females***	***Males***	

Lack of extension	3	10	0.62
Complete extension	2	15	

***ROM***	***Females***	***Males***	

90 degrees or less	3	7	0.3
At least 120 degrees	2	18	

***Stability***	***Females***	***Males***	

Abnormal stability	0	1	1.0
Normal stability	5	24	

* = Significant difference.

As the number of observations in age groups, surgical procedures, and comorbid conditions was few in some categories, it was impractical to apply logistic regression analysis to assess their relationship to functional outcomes.

## DISCUSSION

In the current research, three-month follow-up results showed that tibial plateau fractures treated with ORIF and external fixator have significant functional outcome differences between genders. Males recovered better than females at a three-month follow-up (p=0.02, OR=0.05). After three months of treatment, males achieved 92% excellent-good outcomes while females 8%. Similar findings regarding the influence of gender on functional outcomes were found in a study conducted by Hassan HU.^20^ The study included one hundred closed tibial plateau fracture patients with a mean age of 37±9 years treated with the Ilizarov procedure out of which 70% were males and 30% were females. At three months follow-up, the authors reported that 77% of the males achieved excellent functional outcomes compared to 73% of the females with significant results (*p=*0.016).^20^

In the present findings at three months follow-up, individual components of the functional scoring scale showed significantly higher pain in females than males. Although non-significant, other functional capacities like knee extension, range of motion, and walking capacity were better in males than females. One possibility of functional outcomes difference in gender is previous reports on higher pain sensitivity as well as post-surgical pain scores and pain events in females compared to males.^25^,^26^ Notably, in ankle fracture surgery, females have been reported to have higher postoperative pain scores.^27^ Other studies also observed males to have a higher pain threshold level. Moreover, there are racial variations in pain threshold.^28^ Another possibility is that some difference in the anatomy of the tibial plateau between genders might also influence functional outcomes and recovery after fracture treatment. Research studies conducted on proximal tibia morphological measurements have showed significant differences in plateau aspect ratio between genders. Females have smaller dimensions and a greater patella-tibial Q angle than males. 29,30 Research studies found no geometrical difference between genders. 31 Present findings suggest the combined difference in pain response and normal anatomical differences between genders potentially affect functional outcomes.

Concerning age, the functional outcome in a study showed statistically better functional outcomes in younger patients as compared to the elderly (*p=*0.017).^20^ In the current research, the Fisher Exact test showed a non-significant difference between functional outcomes to age groups (*p*>0.05). Our findings support the other available data according to which patients of all age groups respond equally well to both external fixation and ORIF procedures.^15^-^19^,^21^,^23^ However, these studies also do not support current research findings on gender. In one study, 153 tibial plateau fracture patients were divided into two age groups of less than or greater than 60 years of age.^15^ In another study, 96 tibial plateau fracture patients had a very similar manner of age grouping as our research (18-41 years, 42-51 years, 52-63 years, and 64-94 years).^19^ Both studies concluded that older age is not a risk factor for poorer outcomes after ORIF and that ORIF is as effective in the elderly as it is in younger patients. Thus, this supports our results on different age groups of tibial plateau fracture patients treated with ORIF and external fixators.

Current research findings showed a non-significant difference in functional outcomes between ORIF and External fixator procedures at all follow-up visits (p>0.05). Some of the retrospective studies compared the functional outcomes of patients after both ORIF and external fixation. One such study was conducted by Chan C that included a total of 58 tibial plateau fracture patients out of which 24 were treated with ORIF and 35 with external fixation. At a minimum of two years of follow-ups, there was no statistically significant difference in the outcomes assessed by the Rasmussen Score for both the ORIF and external fixation procedures.^22^

In another study, 36 patients were treated with ORIF and 28 with external fixators. A non-significant difference was observed between both procedures at a mean follow-up time of 43±10.8 months.^23^ These studies support our research findings which suggest that functional outcomes are not affected by the ORIF, and external fixator surgical approaches used to manage tibial plateau fractures. Contrary to this, in complex tibial plateau fractures, it is reported that an external fixator has better functional outcomes compared to the ORIF procedure.^32^

In our study, it was observed that the gender difference in outcome scores was mainly in the pain component of the scoring system. Thus, our data provides novel insight into gender-specific pain perception in people belonging to different races in Pakistan.

### Limitations:

This is a single-center observational study with an inadequate and unequal sample size in groups that might overestimate the effect size. Thus, there are limitations in terms of generalizability and drawing explicit conclusions on comparing gender outcomes. To generate accurate results, future studies on larger sample sizes are warranted.

## CONCLUSION

Functional outcomes of patients with tibial plateau fractures are not likely to be affected by the ORIF or external fixator procedures, the patients respond almost equally to both. Age also does not influence functional outcomes. However, potentially males have a better functional outcome at three months post-procedure compared to females, mainly in the pain component, indicating that gender differences possibly affect the functional outcomes of tibial plateau fractures.

This finding might facilitate orthopaedic surgeons in predicting tibial plateau fracture treatment outcomes in genders and developing better treatment strategies for the timely restoration of functional capacity in all patients equally. Hence, achieving sustainable goals in better understanding of existing patient problems and their need-based treatment. Further exploration of variations in gender-based fracture management response possibly builds on the findings of this study and influences surgeon’s treatment plans and approaches.

### Author contribution:

**TA:** Contributed to the study design, questionnaire design, data interpretation, and provided feedback through critical manuscript review

**ZAM:** Contributed to the study concept, study design, literature search, data collection, analysis, and interpretation

**FZ:** Contributed to the literature search and manuscript writing.

All authors are responsible and accountable for the accuracy and integrity of the work.

## References

[1] Elsoe R, Larsen P, Nielsen NP, Swenne J, Rasmussen S, Ostgaard SE (2015). Population-Based Epidemiology of Tibial Plateau Fractures. Orthopedics.

[2] Malik S, Herron T, Mabrouk A, Rosenberg N (2023). Tibial Plateau Fractures 2023 Apr 22. StatPearls [Internet].

[3] Mustonen AO, Koivikko MP, Lindahl J, Koskinen SK (2008). MRI of acute meniscal injury associated with tibial plateau fractures:prevalence, type, and location. Am J Roentgenol.

[4] Rademakers MV, Kerkhoffs GMMJ, Sierevelt IN, Raaymakers ELFB, Marti RK (2007). Operative Treatment of 109 Tibial Plateau Fractures:Five- to 27-Year Follow-up Results. J Orthop Trauma.

[5] Elsoe R, Johansen MB, Larsen P (2019). Tibial plateau fractures are associated with a long-lasting increased risk of total knee arthroplasty a matched cohort study of 7,950 tibial plateau fractures. Osteoarthritis Cartilage.

[6] He QF, Sun H, Shu LY, Zhan Y, He CY, Zhu Y (2018). Tibial plateau fractures in elderly people:an institutional retrospective study. J Orthop Surg Res.

[7] Schatzker J, McBroom R, Bruce D (1979). The tibial plateau fracture. The Toronto experience 1968--1975 Clin Orthop Relat Res.

[8] Müller ME, Nazarian S, Koch P, Schatzker J (2012.). The comprehensive classification of fractures of long bones:Springer Sci Business Media.

[9] McNamara IR, Smith TO, Shepherd KL, Clark AB, Nielsen DM, Donell S (2015). Surgical fixation methods for tibial plateau fractures. Cochrane Database Syst Rev.

[10] Papagelopoulos PJ, Partsinevelos AA, Themistocleous GS, Mavrogenis AF, Korres DS, Soucacos PN (2006). Complications after tibia plateau fracture surgery. Injury.

[11] Ramos T, Ekholm C, Eriksson BI, Karlsson J, Nistor L (2013). The Ilizarov external fixator--a useful alternative for the treatment of proximal tibial fractures. A prospective observational study of 30 consecutive patients. BMC Musculoskelet Disord.

[12] Naja AS, Bouji N, Eddine MN, Alfarii H, Reindl R, Tfayli Y (2022). A Meta-analysis Comparing External Fixation against Open Reduction and Internal Fixation for the Management of Tibial Plateau Fractures. Strategies Trauma Limb Reconstr.

[13] Hall JA, Beuerlein MJ, McKee MD (2009). Open reduction and internal fixation compared with circular fixator application for bicondylar tibial plateau fractures. Surg technique. J Bone Joint Surg Am.

[14] Wang Z, Zheng Z, Ye P, Tian S, Zhu Y, Chen W (2022). Treatment of tibial plateau fractures:A comparison of two different operation strategies with medium-term follow up. J Orthop Translat.

[15] Kim JK, Hwang KT, Soh HS, Shon OJ, Park KC (2022). Comparison of tibial plateau fracture surgical outcomes between young and elderly patients:are outcomes really poorer in the elderly?. Arch Orthop Trauma Surg.

[16] Maseda M, Konda S, Leucht P, Ganta A, Karia R, Egol K (2023). Tibial plateau fractures in the elderly have clinical outcomes similar to those in younger patients. Eur J Orthop Surg Traumatol.

[17] Dekhne MS, Stenquist D, Suneja N, Weaver MJ, Petersen MM, Odgaard A (2022). Outcomes after ORIF of Bicondylar Schatzker VI (AO type C) Tibial Plateau Fractures in an Elderly Population. Injury.

[18] Oladeji LO, Worley JR, Crist BD (2020). Age-Related Variances in Patients with Tibial Plateau Fractures. J Knee Surg.

[19] Van Dreumel RL, Van Wunnik BP, Janssen L, Simons PC, Janzing HM (2015). Mid- to long-term functional outcome after open reduction and internal fixation of tibial plateau fractures. Injury.

[20] Hassan HU (2022). Functional Outcome of Ilizarov Technique in Managing Proximal Tibial Fracture in Combined Military Hospital, Rawalpindi. J Islamabad Med Dent Coll.

[21] Mondal T, Ghosh S, Ghosh S (2022). Study of surgical and functional outcome in the management of proximal tibial fracture with circular wire-based external fixation. Hamdan Med J.

[22] Chan C, Keating J (2012). Comparison of outcomes of operatively treated bicondylar tibial plateau fractures by external fixation and internal fixation. Malays Orthop J.

[23] Oguzkaya S, Misir A, Kizkapan TB, Eken G, Ozcamdalli M, Basilgan S (2022). A comparison of clinical, radiological, and quality-of-life outcomes of double-plate internal and Ilizarov external fixations for Schatzker type 5 and 6 tibia plateau fractures. Eur J Trauma Emerg Surg.

[24] Rasmussen PS (1973). Tibial condylar fractures impairment of knee joint stability as an indication for surgical treatment. J Bone Joint Surg Am.

[25] Tighe PJ, Riley JL, Fillingim RB (2014). Sex Differences in the Incidence of Severe Pain Events Following Surgery:A Review of 333,000 Pain Scores. Pain Med.

[26] Mogil JS (2012). Sex differences in pain and pain inhibition:multiple explanations of a controversial phenomenon. Nat Rev Neurosci.

[27] Storesund A, Krukhaug Y, Olsen MV, Rygh LJ, Nilsen RM, Norekvål TM (2016). Females report higher postoperative pain scores than males after ankle surgery. Scand J Pain.

[28] Dawson A, List T (2009). Comparison of pain thresholds and pain tolerance levels between Middle Easterners and Swedes and between genders. J Oral Rehabil.

[29] Lim HC, Bae JH, Yoon JY, Kim SJ, Kim JG, Lee JM (2013). Gender differences of the morphology of the distal femur and proximal tibia in a Korean population. Knee.

[30] Hsu RW, Himeno S, Coventry MB, Chao EY (1990). Normal axial alignment of the lower extremity and load-bearing distribution at the knee. Clin Orthop Relat Res.

[31] Yue B, Varadarajan KM, Ai S, Tang T, Rubash HE, Li G (2011). Gender differences in the knees of Chinese population. Knee Surg Sports Traumatol Arthrosc.

[32] Tripathy SK, Varghese P, Panigrahi S, Panda BB, Srinivasan A, Sen RK (2021). External fixation versus open reduction and internal fixation in the treatment of Complex Tibial Plateau Fractures:A systematic review and meta-analysis. Acta Orthop Traumatol Turc.

